# Unlocking the potential: laser surface modifications for titanium dental implants

**DOI:** 10.1007/s10103-024-04076-1

**Published:** 2024-06-24

**Authors:** Bela Kolarovszki, Szabolcs Ficsor, Dorottya Frank, Krisztian Katona, Balazs Soos, Kinga Turzo

**Affiliations:** Dental School, Medical Faculty, University of Pécs, Tüzér u. 1, Pécs, 7623 Hungary

**Keywords:** Dental implant, Titanium, Osseointegration, Biointegration, Laser surface modification, Roughness

## Abstract

The review critically evaluates the current state of studies investigating laser irradiation for modifying titanium surfaces to enhance the biointegration of dental implants. Laser modification is a rapidly evolving physicochemical surface modification process with the potential to revolutionize dental implant technology. A thorough search of electronic databases, including PubMed, Science Direct, MEDLINE, and Web of Knowledge, was conducted to identify relevant articles. The review focuses on the surface features of laser-modified implants, encompassing in vitro cell culture experiments, rare animal experiments, and limited clinical trials. Of the 26 selected sources, 21 describe surface features, while only two involve in vivo human experiments. The review highlights the lack of long-term clinical experience and calls for further research to mature these technologies. Despite the absence of a consensus on optimal laser types and settings, the overall results are promising, with few negative outcomes. As research in laser irradiation of titanium surfaces progresses, significant advancements in dental implant technology and improved patient well-being are anticipated.

## Introduction

The management of partial or total edentulism presents a complex challenge for dental practitioners globally. In response, contemporary dentistry has embraced implantation as a paramount solution. Nevertheless, a notable cohort of patients exhibits insufficient bone volume or quality necessary for successful implantation, notwithstanding their preference for immediate loading of implants. Consequently, dental professionals and the industry persistently endeavor to innovate, striving to surmount these impediments and align with patient expectations.

Both oral and orthopedic surgery rely on dental and medical implants primarily crafted from titanium and its alloys. The successful biointegration of these implants into host tissue predominantly hinges on their surface chemical composition and morphology. Predominantly, interactions between the implant and biological tissue manifest at the bone-implant interface. To foster biointegration, various surface modification processes have been developed, categorized into physicochemical, biochemical, and morphological modalities [1]. 

The primary objective is to retain the implant’s fundamental physical properties while selectively altering its outermost surface to regulate its physiological interaction. Although numerous surface modifications remain in the experimental phase, prospective in vitro, in vivo, or clinical studies are anticipated. Favorable outcomes hold particular significance for elderly or infirm patients [2]. 

Physicochemical treatments encompass chemical surface reactions (e.g., oxidation, acid-etching), sandblasting, ion implantation, laser ablation, and coating with inorganic calcium phosphate. These methodologies aim to modulate the surface’s energy, charge, and composition, thereby imparting diverse roughness and morphology [1]. 

In recent years, laser-based techniques have garnered increasing attention for augmenting surface roughness. Key advantages of lasers encompass precise frequency control, a broad frequency spectrum, high energy density, focused and rastered light, and the capacity for pulsating the source to regulate reaction times. Laser application for surface modification to enhance biocompatibility encompasses various approaches, with surface cladding and alloying being predominant. Laser surface remelting processes, characterized by simplified procedures with fewer process parameters, demonstrate potential advantages in industrial settings [3]. 

The fundamental prerequisite for the uneventful healing of implants lies in osseointegration, influenced partly by the patient’s health, surgical technique, type of dental implant employed, and its material and surface attributes [4]. 

The enduring success and functional efficacy of titanium and its alloys have established them as the gold standard of implantology. Periodically, endeavors are made to develop novel endosseal implants utilizing alternative metals such as gold, stainless steel, or cobalt chrome alloys. However, unfavorable tissue reactions and suboptimal success rates impede the widespread adoption of these materials for clinical applications. Consequently, titanium (Ti) and its alloys, alongside more recently introduced zirconia (Zr), emerge as the most frequently utilized and reliable alternatives [5]. 

Titanium, constituting the 9th most abundant element in the Earth’s crust and the 4th most common metal, exhibits versatility in alloy formation with various metals, thereby enabling the enhancement of its properties. Among these, the Ti-6Al-4 V alloy is most prevalent, composed of 90% titanium, 6% aluminum, and 4% vanadium [6]. 

This alloy manifests an alpha-beta crystalline structure, wherein aluminum contributes to alpha phase stabilization, enhancing strength and reducing weight, while vanadium stabilizes the beta phase. Titanium and its alloys boast advantageous chemical and mechanical properties, characterized by low material density (approximately 60% of steel’s density) coupled with hardness, strength, and toughness. Moreover, their thermal expansion coefficient is lower than that of steel, with an elastic modulus closely resembling that of natural bone, surpassing other metals employed in implantation. Notably, these materials develop a protective oxide layer upon exposure to air, commencing within milliseconds, which also forms under physiological conditions within tissue. This stable oxide layer, predominantly comprising TiO2, confers titanium’s exceptional biological inertness, shielding it from undesired corrosion and galvanization in the oral environment [6]. 

Biological tissue predominantly interacts with the outermost atomic layers of an implant, particularly within a 0.1 to 1 nm thickness [7], crucial for controlling the bone-implant interface. To optimize this interface, various surface modification methods have been developed, categorized into physicochemical, biochemical, and morphological alterations [8]. 

## Physicochemical surface modifications

Surface tension (in other words the wettability of the surface), charge and material composition are the distinctive properties we try to modify with physicochemical techniques. Techniques such as: sandblasting; acid-etching; anodization (oxidation); ion-implantation; coatings on the surface and lastly melting and ablating layers of material using lasers [1, 2]. 

Calcium-phosphate coatings have been studied extensively because they closely resemble the minerals found in bone [9]. Titanium oxides adsorb Ca^2+^ and PO_4_^2−^ ions from physiological liquids. Therefore, in theory, the titanium surface becomes like calcium-phosphate as time progresses. However, when used as coating calcium-phosphate presents itself as a better focal point for the creation of crystals in a similar phase from the ion content of tissue fluid. Another frequently applied coating are hydroxyl-apatite crystals. A positive outcome of these coatings is the inhibition of the dissolution of titanium ions and their dispersion into the environment. Often these coatings can be produced using an ion spraying technique. The drawback of these technologies is that the retention of the coating is often insufficient, especially if the implant is under load. Because of this the coating can separate from the metal surface and the whole retention of the implant can be jeopardized [10]. 

Ion-implantation can be used to improve the implants mechanical properties. For example, implanting iridium or nitrogen into Ti6Al4V alloy the implant’s corrosion resistance can be improved. Combining different physicochemical processes can result in compounded improvement for the material. This is true for example in the case of SLActive implants from the Straumann company. SLA is the abbreviation of sand blasted – acid -etched. SLActive implants are also a successful example for the importance of surface hydrophilicity. After sand blasting and etching this implant is neutralized in nitrogen atmosphere and is stored in NaCl solution. This results in exceptionally great hydrophilicity, and so the adsorption of different proteins from tissue fluid is faster, osseointegration improves and the implant’s stability gets better in the critical 2–4 weeks after surgery [2]. 

### Biochemical surface modifications

Biochemical processes are an alternative to physicochemical processes. Biochemical surface modifications alter the biology and biochemistry of implantation and the participating cells’ function and differentiation. In contrast to the inorganic coatings, these techniques utilize organic compounds. Different peptides, enzymes and proteins are imparted to the interfacial surface, sometimes they are also immobilized. Using cell adhesion molecules is a major way to influence the relationship between the alloplastic material and tissue. Since the discovery that peptide chains containing Arg- Gly- Asp (RGD) sequences can mediate cells’ affinity towards different matrix proteins (for example: fibronectin, type I collagen, osteopontin, etc.) studies are being conducted to bring these peptides to implant surfaces. Cell surface receptors of the integrin superfamily can recognize the RGD sequences and can trigger cell adhesion [11]. 

A different approach to surface modification is the allocation of growth factors to the implant surface. Two major factors decide the efficacy of these substances: (1) biomolecules must retain interaction with cells and (2) locally biomolecules must reach an appropriate threshold concentration. These factors can be controlled if we immobilize the molecules to the surface or allow them to be released from coatings. The simplest method is to immerse the implants into a solution of proteins and thus the proteins are adsorbed to the surface. A drawback of this method is we cannot control the release or the orientation of molecules. Moreover, the weak secondary bonding can further weaken due to a change in the environment. A bit more complicated method is immobilization. Utilizing hydroxyl groups on the surface of titanium oxides a more powerful and more durable bond can be created between metal and protein [1]. A similar technique is the creation of polyelectrolyte (PE) coatings. To create such coatings alternating layers of polycations and polyanions must be imparted on the implant surface. These coatings can also be used to influence the adsorption of bioactive proteins. Furthermore, if these multi-layered coatings contain signal transmitting molecules, then local cell responses could be controlled [12].

### Morphological surface modifications

This represents the third primary classification of modifications. These processes are frequently intertwined with physicochemical alterations, making it challenging to draw a clear line of demarcation between them. However, they share a common characteristic: the absence of bioactive molecules or any significant change in the chemical structure of the surface. Instead, the focus lies solely on reshaping the surface. Through these alterations in shape, micro- and macro-mechanical retention is established between the implant and its surrounding environment. Furthermore, the intriguing phenomenon of “contact guidance” manifests itself in these modifications. The most important property of surface morphology is surface roughness (R_a_). Higher roughness is advantageous in comparison to polished surfaces, for the surface is greatly expanded due to the microstructure. It has become clear that epithelial cells do not adhere to rough surfaces as much as they do to polished surfaces (R_a_ < 0.5 μm). In contrast, fibroblast do not seem to have this preference [13]. In case of high R_a_ the metabolic activity of osteoblast like cells is increased. They produce more osteocalcin, growth factor (TGF- β1) and alkaline phosphatase. Since these substances are also the biomarkers for bone growth, we can conclude that higher surface roughness is beneficial for ossification.

The aim of this paper was to create a literature review about the fastest evolving physicochemical surface modification process: laser modification. These modifications are versatile and provide precise and contact free manufacturing. Lasers can be grouped like so: the wavelength of light leaving the device, the output, if the device is operating in continuous or pulsed mode, the state of matter of the laser-medium, and the method of pumping. Lasers operate by the phenomenon of population inversion and stimulated emission. To create the laser, beam a laser-medium, and a way to excite this medium, is required. During excitation a state of population inversion occurs among the particles of the medium. This process is called pumping. Laser-media can be gas (excimer laser for example), liquid or solid (e.g. Nd: YAG lasers). Among solid matter media semiconductors represent a subgroup. Lastly 3 different methods of pumping exist: chemical, electronic or optical [14]. 

It is also found that the direct femtosecond laser processing can produce novel materials with wetting properties ranging from super hydrophilic to super hydrophobic. The super wicking effect produced through this technique can be so strong that the treated surfaces can make liquids run vertically uphill over an extended surface area. The super wicking effect has been demonstrated on a range of solid materials, including metals, silicon, glass, and biological hard tissues. Furthermore, several studies have shown that the femtosecond laser surface nano/microstructuring has a potential to produce biomaterials with superior functionality [15]. 

## Method

The following sources were used for the study: PubMed, MedLine, Google Scholar and standard Google searches. Keywords (implant, titanium, laser, surface modification, roughness, and biocompatibility, along with their combinations) were used to conduct the searches, highlighted on dental sciences. Additionally, any relevant sources cited in the reviewed literature were also considered. In the first step 45 publications were initially selected.

After a comprehensive examination of these publications, a careful evaluation led to the inclusion of only 26 of them in this paper. The primary focus of the selected works centered around the morphological and physicochemical surface modifications of titanium dental implants. While other surface modification techniques were summarily mentioned in the introduction, they were not explored in-depth within this study.

## Results and discussion

Chronological order was used for the reviewed publications, the most recent ones were listed first. Each publication highlights the type of laser used, the research circumstances and the obtained results. To ensure transparency and easy reference, all this information is compiled in a spreadsheet located at the end of this paper. This approach allows for a clear and organized presentation of the findings, promoting a better understanding of the research landscape surrounding morphological and physicochemical surface modifications of titanium dental implants through laser applications.

Khoo et al. [16] concluded in their review that laser-modified implants could be successful alternatives to commonly used SLA and anodized forms. While long-term human and clinical trials (> 5 years) are lacking, animal experiments showed laser-modified implants outperforming machined or SLA implants in removal torque measurements 1 to 6 weeks after insertion into the bone. The study suggests lasers can create contamination-free titanium implants with thick oxide layers, promoting enhanced osseointegration compared to conventional methods. Laser surface modification holds promise for improving titanium dental implant performance in implantology.

Tiainen et al. [17] (see Table 1.) used Nd: YAG laser to create grid-like structures on titanium alloy discs, investigating their impact on wettability and friction during bone insertion. Laser parameters remained fixed, and patterns were formed by three-dimensional laser movement. The grid structure, composed of micrometer-scale fissures, varied in thickness (40 μm to 140 μm) and showed improved water repellency over sandblasted samples. Despite initially demonstrating super-wettability, cleaning removed this property, revealing a coating of nanometer-sized particles. Friction tests on bovine bone indicated increased friction for all laser-modified discs compared to controls, with the 40 μm fissures exhibiting optimal properties, potentially enhancing implant stability after insertion.

Hindy et al. [18] reviewed 28 publications on laser-modified Ti implant surfaces using in vitro cell cultures, mainly osteoblasts. Analyzing morphology, adhesion, vitality, proliferation, and differentiation, they used tetrazolium bromide (MTT), Alamar Blue, and cell counting. Nd: YAG laser on Ti6Al4V alloy was most common; only 9 studies used commercially pure titanium (CpTi). Due to varied lasers and parameters, statistical analysis was impossible, but several conclusions emerged. Most laser modifications improved or maintained cell adhesion/growth compared to other methods. Laser type, wavelength, and parameters influenced cell culture development. Importantly, laser modification increased surface oxide layer thickness, and structures similar in size to cells positively impacted vitality and growth.

Zwahr et al. [19] (see Table 1.) used direct laser interference patterning (DLIP) to create a microstructure on titanium surfaces with parallel lines. The Nd: YAG laser split into two beams, reaching the surface at different angles, determining line spacing (5 μm, 10 μm, 20 μm). Laser energy density influenced structure depth and shape. Lower density resulted in melted metal flowing and solidifying with sharp edges. Higher density caused pooling of melted metal, while further increases led to ablation. SEM confirmed degradation of the regular structure. Laser-modified surfaces exhibited a 20% wettability increase, attributed to morphology and elevated nitrogen content (5x compared to control). Osteoblast cell vitality increased by 16%, with a moderate cell count increase.

Studies, including those by Cunha et al. [20] (see Table 1.) and Lang et al. [21], identify peri-implantitis as a major cause of implant loss, characterized by bacterial inflammation leading to gradual bone loss. Staphylococcus aureus, a permanent oral microbiome member, is a key pathogen. Cunha et al. created laser-modified titanium surfaces with reduced bacterial adhesion, aggregation, and biofilm accumulation compared to polished surfaces. Using a Yb: KYW laser with 1030 nm wavelength and 500 fs pulses, the modified surface featured an Ra of 0.3 μm, comprising fissures and nano-spikes smaller than bacterial size. Enhanced wettability did not increase bacterial adhesion, contrary to previous theories. Titanium’s antibacterial property is influenced by morphology and composition, specifically the width of oxide layers and the proportion of anatase (TiO2) crystalline form. Both increased in the experiment, contributing to the antibacterial effect. However, even the control titanium implant with inferior properties exhibited significant oxides and anatase, suggesting that surface structures play a more decisive role in the antibacterial effect.

Lee et al. [22] (see Table 1.) examined carcinoma and fibroblast cell proliferation and adhesion on laser-modified titanium surfaces. They created 5 μm-wide indentations on polished titanium with 15 μm and 30 μm spaces, aiming to enhance epithelial and connective tissue attachment without significantly altering plaque accumulation. In vitro culturing and fluorescent dyeing showed no significant change in cell numbers, but improved epithelial cell attachment, increased adhesion protein production, and larger cells were observed.

Farronato et al. [23] (see Table 1.) conducted a clinical study comparing Laser-Lok and machined implants in 77 patients. Laser-Lok implants, with microscopic channels on the neck, demonstrated greater clinical attachment levels (CAL) and roughly halved peri-implant crestal bone loss (CBL) compared to machined implants after 6, 12, and 24 months.

Györgyey et al. [24] (see Table 1.) performed the first cell culture experiment on laser-modified titanium implants. KrF or Nd: YAG laser treatment on SLA disks reduced surface roughness, with Nd: YAG causing micro cracks. Despite damaged TiO2 layers, cell growth was not significantly different from untreated disks, supporting the idea that surface morphology, not the oxide layer, influences cellular response.

Ketabi and Deporter’s 2013 study [25] reviewed literature on the relationship between periodontal tissue and micrometer fissures created by lasers on titanium implant necks. Laser-ablated surfaces, distinct from traditionally machined and polished surfaces, showed a significant reduction in bone loss near the implant. Laser modification not only minimized bone loss but also allowed for direct attachment of periodontal ligaments, acting as a barrier against apical migration of epithelial cells and potentially mimicking natural supportive tissue.

Wittig et al. [26] (see Table 1.) utilized photolithography to create micrometer-sized surface structures and examined their impact on osteosarcoma cell proliferation and attachment. The unique process, featuring a high three-dimensional resolution, resulted in a pattern of columns and rods coated with TiO2 for enhanced biocompatibility. Osteosarcoma cells exhibited enhanced spreading in three dimensions compared to polished controls, emphasizing the significance of 3D shapes in influencing cell differentiation during osteogenesis.

Celen and Özden [27] (see Table 1.) used a Q-switched Ytterbium fiber laser to create surface textures on implant materials, aiming to distribute forces more evenly to the bone. Different textures, including honeycomb, diamond, sphere, and moon shapes, were examined for their impact on force distribution through optical microscope, SEM, and finite element analysis. The honeycomb shape showed promising results, suggesting that laser-created surface formations could act as a quasi-artificial periodontal ligament structure, improving force distribution in implants.

Man et al. [28] (see Table 1.) employed Nd: YAG laser to create surface textures on CP titanium and TiN-coated titanium, featuring microscopic dimples. TiN coating, formed through laser irradiation in a nitrogen atmosphere, reduced corrosion and allowed for the creation of interconnected subsurfaces under the dimples. The authors hope this structure facilitates deeper migration of bone cells and better implant retention due to improved nutrient circulation.

Nevins et al. [29] (see Table 1.) investigated the relationship between laser-modified surfaces and epithelial cells and periodontal fibers. Laser ablation was used to create regular channels on titanium implant necks. The study aimed to address crestal bone loss, a common issue caused by inflammation and apical migration of epithelial cells. Laser-Lok implants were inserted into patients’ jawbones, and after 6 months, examination revealed connective tissue fibers at an oblique angle toward the laser-modified surface, potentially preventing unwanted bone loss. While promising, the study’s small sample size and absence of oral cavity loading warrant cautious interpretation.

Faeda et al. [30] (see Table 1.) implanted 48 CP titanium implants into rabbits, with 24 modified by Nd: YAG laser. The modified implants showed significantly higher Removal Torque (RTQ) in all healing periods (4, 8, 12 weeks) compared to control. Control implants showed time-dependent anchorage creation, requiring less force for removal in the initial 8 weeks, while forces for modified implants increased continuously.

Braga et al. [31] (see Table 1.) utilized Nd: YVO4 laser to create distinct structures on CP titanium surfaces, examining their impact through SEM and X-ray diffraction. Laser parameters affecting energy density, frequency, and scanning speed resulted in various surface textures. The study emphasized that, for surface texturing, energy density alone is insufficient; scanning speed, area, and frequency of irradiation, and scanning motion velocity are crucial factors.

Vorobyev et al. [32] (see Table 1.) explored the surface-modifying effect of a Ti: sapphire laser in the femtoseconds range on titanium disks. Different energy densities and impulses were used, resulting in various surface structures. The study demonstrated that femtosecond lasers, applying different parameters, can create formations different in size and appearance compared to other lasers.

Mirhosseini et al. [33] (see Table 1.) laser-modified Ti6Al4V alloy disks, creating three different surface structures. Surface roughness increased, contrary to the expected increase in contact angle. MTT analysis showed increased cell count, with the least rough surface exhibiting the highest increase, suggesting an optimal surface roughness for cell proliferation.

Hao et al. [34] (see Table 1.) studied the wettability of Ti6Al4V alloy after laser modification, observing decreased droplet contact angles and improved wettability with increasing energy density. The study also investigated laser modification’s effects on osteoblast cell adhesion, proliferation, and apatite crystallization in simulated body fluid (SBF), showing positive outcomes for cell behavior.

Kurella et al. [35] reviewed CO2, Nd: YAG, and excimer lasers, highlighting their characteristics and applications. CO2 lasers have high energy intensity, but they may increase surface oxidation and create fragile rims. Nd: YAG lasers offer controlled impulses and operate at lower wavelengths for focused surfaces. Excimer lasers, utilizing high-energy dimers, generate UV laser beams for high-resolution surface microtextures with low thermal effects.

Karacs et al. [36] (see Table 1.) inserted titanium implants into rabbits with different modified surfaces, including machined, sand-blasted, and laser-modified samples. After 3 months, the sand-blasted sample showed the best osseointegration, while laser-modified samples exhibited decreased roughness and increased Removal Torque (RTQ), suggesting improved osseointegration despite lower roughness.

Cho et al. [37] (see Table 1.) created honeycomb-like structures on CP titanium implants and observed a significant increase in RTQ after laser modification. The RTQ more than tripled from 23 Nm to 63 Nm after 8 weeks of healing in rabbit tibiae.

Bereznai et al. [38] (see Table 1.) used excimer lasers on titanium disks to either polish or increase roughness. Ten impulses of an ArF laser significantly reduced roughness to inhibit plaque accumulation. Shorter wavelength picosecond KrF excimer laser avoided undesirable formations, ensuring a clean surface.

Pető et al. [39] (see Table 1.) studied laser ablation effects on four surfaces: machined, sand-blasted, and two laser-modified. Laser-modified surfaces showed unique morphology in the 10 μm range, eliminating contamination and leaving a clean surface.

Hallgren et al. [40] (see Table 1.) examined osseointegration of initially machined titanium implants laser-modified by Nd: YAG. Laser-modified implants with grid-like pits showed increased interfacial surface and RTQ compared to unmodified samples. Material composition changes were minimal, with laser-modified implants possibly having greater micromechanical retention and reduced movement stimulus on surrounding tissue.

Gaggl et al. [41] (see Table 1.) compared the morphology and purity of laser-modified titanium implants. Optimal surface roughness was previously determined as 11.6 μm distance between two surface microstructures and an average height difference of 1.4 μm [40]. Plasma-sprayed and laser-modified samples approached these values, but plasma-sprayed surfaces exhibited residual contamination (mostly Na and S), while laser-modified and machined samples showed high purity in EDS analysis. Sand-blasting with aluminum oxides and fluoric acid etching increased Ra, but resulted in less optimal structure and higher levels of Al contamination.

Research on laser-modified dental implants has unveiled critical insights. Since the advent of intraoral implants in the 1960s, considerable attention has been devoted to surface modifications, driven by advancements in understanding the molecular and cellular processes at the interface. While lasers represent a contemporary advancement, their application is relatively recent and still harbors uncertainties. Most studies elucidate precise modifications and conduct in vitro experiments, with limited involvement of animal and human trials. Among 26 sources reviewed, 21 underscore surface features, with only two involving in vivo human experiments, indicating a disparity between research and clinical trials.

**Table 1:** Summary table in descending chronological order, highlighting the applied laser, the surface examination methods and the type of experiments

	Author	Applied laser type			Applied surface examination method				In vitro examination			In vivo animal experiment			In vivo human experiment		
1	Tiainen_2019	Q-switched diode pumped Nd: YAG λ = 1064 nm			XPS, EDS, SEM, contact angle, profilometry												
					Optical and confocal microscopy, x-ray diffraction												
					tribometer (friction test on bone surface)												
2	Zwahr_2017	Nd: YAG λ = 532 nm; 2 laser beams			confocal microscopy, SEM, XPS				Osteoblast cell culture, vitality examination WST-1,								
					contact angle				Cell counting, fluorescent microscopy								
3	Cunha_2015	Yb: KYW λ = 1030 nm 500 fs			AFM, SEM, XPS, x-ray diffraction, micro-Raman spectroscopy												
4	Lee_2015	Ti: sapphire λ = 800 nm 220 fs			SEM, optical microscopy				YD-38 and MRC-5 cell culture:								
									Fluorescent microscopy								
5	Farronato_2014	(LaserLok implant)													Randomized clinical trial		
															on 77 patients		
6	Györgyey_2013	Nd: YAG λ = 532 nm		KrF excimer λ = 248 nm	SEM, AFM, XPS				MG-63 osteoblast cell culture: MTT, AB, ALP								
7	Wittig_2012	Ed fiber laser λ = 780 nm 150 fs			SEM				SaOs-2 osteosarcoma cell culture:								
									Fluorescent microscopy								
8	Celen_2012	Yd fiber laser λ = 1060 nm			SEM, EDS, optical microscopy, *in silico* simulation												
9	Man_2010	Nd: YAG λ = 1060 nm			XRD, SEM, optical microscopy												
10	Faeda_2008	Yd fiber laser λ = 1064 nm			SEM, EDS, digital profilometry							Rabbit tibia implantation: RTQ					
11	Nevins_2008	(LaserLok implant)			µCT, SEM, optical microscopy										4 patients 1–1 implants:		
															implants *en bloc* removed		
12	Braga_2007	Nd: YVO4			SEM, XRD												
13	Vorobyev_2007	Ti: sapphireλ = 800 nm 65 fs			SEM												
14	Mirhosseini_2007	Nd: YAG λ = 1064 nm			Laser profilometry, contact angle SBF				2T3 osteoblast cell culture: MTT								
15	Hao_2005	CO2 laser			SEM, EDS, XPS, XRD, contact angle				Human osteoblast hFOB 1.19 cell culture:								
									Lactate dehidrogenese(LDH) test, MTT								
16	Bereznai_2003	ArF excimer λ = 193 nm; KrF λ = 248 nm 0,5 ps			Optical microscopy, SEM, AFM, XPS, XRD												
17	Karacs_2003	Nd-glass λ = 1054 nm			Optical microscopy, SEM							Rabbit femur implantation: RTQ					
18	Cho_2003	Nd: YAG			SEM, EDS							Rabbit tibia implantation: RTQ					
19	Hallgren_2002	Nd: YAG λ = 532 nm			AES, SEM, laser profilometry, Optical microscopy (histomorphometry)							Rabbit tibia and femur implantation: RTQ					
20	Pető_2002	Nd-glass λ = 1064 nm			SEM, XPS							Rabbit femur implantation: RTQ					
21	Gaggl_1999				SEM, EDS, profilometry												

## Conclusion

Lasers are poised to become more ubiquitous in surface modifications, offering a swift and precise alternative to developing novel surface structures compared to traditional methods. These techniques must be further improved, since medical applications require high accuracy in both mechanical and chemical characteristics.

The non-contact nature, the precision in the wavelength of the light, the wide range of wavelength, the possibility of focusing or widening the light beam of lasers ensures consistent reproducibility, making them a promising avenue for future research and applications.

Despite promising outcomes, further investigation, particularly in animal and human studies, is imperative to fully exploit the potential of laser modification for dental implant integration. Comprehensive long-term clinical data is lacking, necessitating additional research for these processes to mature. Encouragingly, most studies demonstrate positive outcomes, suggesting substantial improvement with laser modification. Ongoing efforts for standardization are in progress, yet further data and guidelines are warranted. Despite the availability of diverse laser equipment, scaling up production volume remains a challenge. Current laser-modified implants, such as CSM Korean and Laser-Lok by BioHorizons, exhibit potential in establishing functional connective tissue attachments, to bonding of oral epithelial cells, and reducing alveolar bone resorption.

## Data Availability

Not applicable.
